# Wolfgang Öchsner, Cornelia Estner, Susanne Kühl: Prüfungen erfolgreich bestehen in den Life Sciences

**DOI:** 10.3205/zma001105

**Published:** 2017-08-15

**Authors:** Elisabeth Schaper

**Affiliations:** 1 Stiftung Tierärztliche Hochschule Hannover, Hannover, Germany

## Recension

Presented here is a useful guidebook to help prepare students for exams in the Life Sciences subject. The guidebook is divided into two sections: the first deals with basic exam preparation and the second specifically discusses Life Sciences exams. The introduction begins with an explanation of specific terminology, such as competence orientation, learning goals, and quality criteria. The compact nature of the terminology review allows the reader to advance quickly into more detailed chapters such as, Time management for exam preparation and Learning for university exams. Each chapter begins with opening questions to be answered within the text and include methods, strategies, exercises, checklists and practical tips for exam preparation. Lastly, each chapter concludes with a summary and references for additional literature of interest. Section two of the guidebook, written in the same structure as the previous section, presents exam formats and legal framework and discusses written, oral and practical exams in detail. The chapter Written examination could have been improved if the formulation rules for the development of multiple-choice questions had been considered for the given examples. Overall, the content's presentation is scientifically based and didactically and logical structured with the intention of supporting life science students with optimal exam preparation. 

Additionally, worksheets can be downloaded from an online toolbox by accessing the link in the table of contents. Unfortunately, the link is indirect and thus the downloadable content can only be accessed through the utb website's search function. The ability to access the content without registration and free of charge is a great benefit of the resource. The pictograms with helpful hints (e.g. exercise, download, tip) are useful in improving the text's overview.

Conclusion: This book is a good companion especially for students who have difficulties in finding a suitable learning strategy, for those who procrastinate, and for students with test anxiety or problems with certain exam formats.

## Competing interests

The author declares that she has no competing interests. 

